# Correlation of tools for objective evaluation of infravesical obstruction of men with lower urinary tract symptoms

**DOI:** 10.1590/S1677-5538.IBJU.2018.0706

**Published:** 2019-09-02

**Authors:** Orestes Mazzariol, Leonardo O. Reis, Paulo R. Palma

**Affiliations:** 1 Universidade Estadual de Campinas - Unicamp, Campinas, SP, Brasil

**Keywords:** Prostatic Hyperplasia, Prostate, Transurethral Resection of Prostate

## Abstract

**Purpose:**

To identify how the most frequently used parameters in daily clinical practice diagnosing bladder outlet obstruction (BOO) due to benign prostate hyperplasia (BPH) correlate to each other.

**Materials and methods:**

The study included 452 patients with lower urinary tract symptoms (LUTS) of the UNICAMP urology outpatient clinic of LUTS. Inclusion criteria: patients with BOO due to BPH who agreed to participate in the study. Exclusion criteria: patients with urinary tract infection, neurological diseases that compromised the lower urinary tract, prior prostatic surgery, radiotherapy or urethral stenosis. Patient assessment: history, international prostate symptoms score (IPSS), nocturnal quality of life score (NQoL) questionnaires, physical and digital rectal examination (DRE), PSA, transabdominal ultrasound with intravesical prostate protrusion (IPP), post-mictional residue and free uroflowmetry.

**Results:**

There was no strong Spearman correlation among the studied variables. The only moderate correlations occurred between IPSS and NQoL (p <0001; c=0.56) and between IPP and prostate volume (p <0001; c=0.57). Weak correlations between IPP and post-mictional residue (p <0001; c=0.31) and free uroflowmetry (p <0001; c=-0.26); and between IPSS and free uroflowmetry (p <0001, c=-0.21) were observed.

**Conclusion:**

In this study, we found moderate, weak, very weak and absent correlation among the various parameters used in the diagnosis and management of BOO due to BPH. As the value of these tools is variable, the creation of a logical and objective algorithm was not possible and the treatment is based on the interpretation of clinical symptoms.

## INTRODUCTION

Lower urinary tract symptoms (LUTS) are frequent in adult males, with an incidence approximately of 40% of those with more than 65 years old. They are consequent of prostate and bladder micturition disorders and include storage symptoms (urgency, frequency, nocturia and incontinence), emptying symptoms (weak stream, urinary exertion, hesitancy and terminal dripping) and post-micturition symptoms (incomplete emptying, post-micturition dripping) (1, 2).

Evaluation of patients with LUTS includes anamnesis, validated questionnaires, physical exam (particularly digital rectal examination-DRE), and auxiliary tests (urine, PSA, ultrasound).

Among all validated questionnaries, the most used is the International Prostatic Symptoms Score (IPSS), that includes eight questions, seven related to symptoms and one related to quality of life (IPSS-QoL). It rates the symptoms as mild (0-7 points), moderate (8-19 points) and severe (20-35 points), and it is an important tool to characterize the severity of symptoms and the follow-up of patients (3).

PSA values above 1.5ng/mL may be related to prostates >30g, with positive predictive value of 78%, and positively correlates to the risk of progression of LUTS (4).

The effect of nocturia on quality of sleep and life may be evaluated by the “Specific Questionnaire of Nocturia and Quality of Life” (NQoL), that includes three domains: sleep/energy (7 questions, scale 0-28), bothersome/worry (5 questions, 0-20 points), and a global question about quality of life (0-4), totalizing 13 items; the question form is self-admnistered , takes 5 minutes to conclude, and proved to be consistent and reproductible (5), although not much used in clinical practice.

While severe LUTS are correlated to prostate anatomy, particularly intravesical protrusion of prostate (IPP), lower quality of life and predispose to inguinal hernia (6, 7), mild LUTS do not linearly correlate with the intensity of symptoms and bladder outlet obstruction; also, progressive benign prostatic hyperplasia causes bladder dysfunction that interferes with the intensity of LUTS, regardless the degree of bladder outlet obstruction (8-11).

Strope et al. showed that in the USA, despite the availability of guidelines proposed by the American Urological Society to treat benign prostatic hyperplasia (BPH), it is observed a great diversity of use and precision of available tools (12).

## OBJECTIVE

To correlate the most frequent parameters used in daily clinical practice to diagnose and treat bladder outlet obstruction (BOO) due to BPH.

## MATERIALS AND METHODS

The study included 452 patients that signed a free informed consent CAAE 84939917.6.0000.5404, during the first interview. All patients attended the ambulatory of Urology of UNICAMP from May, 2018 to September, 2018, complaining of LUTS.

Inclusion criteria: Patients with LUTS that agreed to participate in the study after signing the free informed consent.

Exclusion criteria: Patients with prostate cancer, urinary infection, neurological diseases that affected the urinary system, previous prostatic surgery, radiotherapy or urethral stenosis.

Patient evaluation: Patients were evaluated by history, International Prostatic Symptom Score (IPSS), Nocturnal Quality of Life Score (NQoL), general physical exam and systematized rectal examination (13), serum PSA, transabdominal prostate ultrasound evaluating intravesical prostatic protrusion (IPP), prostatic volume and post-micturition residue, and free uroflowmetry (Qmax) determined by one of the authors (OM). Patients were evaluated before the introduction of drugs in order to avoid bias.

### Statistical analysis

Data were analysed (medium, standard deviation, minimum, maximum, median) and multiple regression models and Spearman Test with Bootstrap method were used to correlate multiple variables among themselves: age, IPSS, IPSS-QoL, NQoL, Qmax, IPP, prostate volume, post-micturition residue and PSA. Significance level was p ≤0.05 and correlation coefficient (c) was classified as very weak (0.00-0.19), weak (0.20-0.39), moderate (0.40-0.59), strong (0.60-0.79) and very strong (0.80-1.00).

## RESULTS

Demographic data of this cohort are summarized at Table-1.

**Table 1:** Characteristics of the studied group.

	Number of observations	Medium±SD	Minimum/Maximum
Age (years)	452	65.8±9.2	30/90
NQoL (Nocturnal quality of life)	452	19±10.3	0/52
IPSS (International prostatic symptoms score)	452	16.2±8.53	0/35
Qol (Quality of life	452	3.02±1.49	1/6
Qmax (maximum flow)	452	10.4±4.8	1/39
Urinated volume	452	155±45.7	16/480
Prostate weight (g)	452	40.9±24.4	7/179
Post-micturition residue (mL)	452	74.3±87.4	0/630
Intravesical protusion of prostate (mm)	452	5.6±5.8	0/33

**Figure 1:**
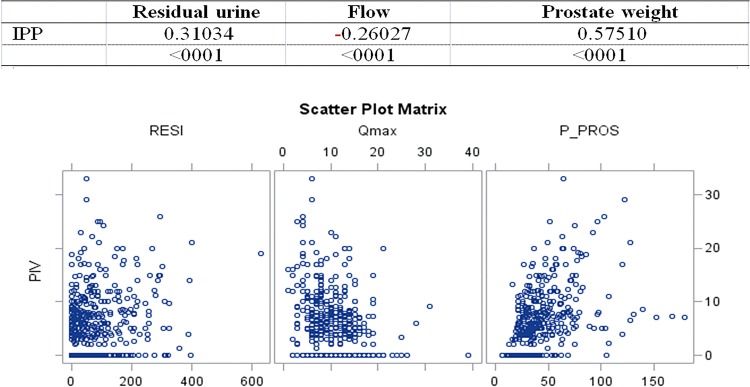
Spearman significant correlations for IPP.

**Figure 2:**
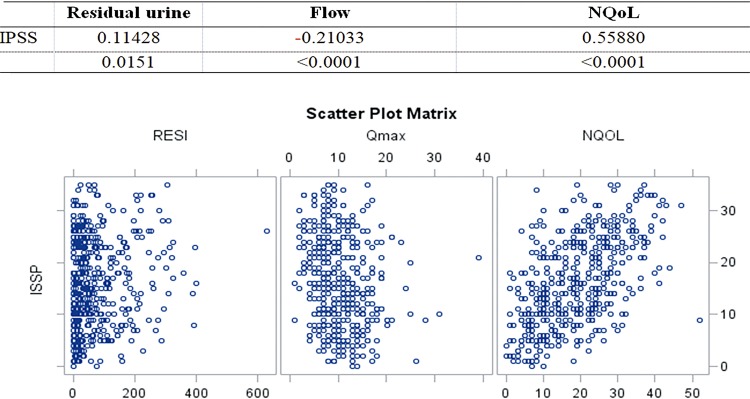
Spearman significant correlations of IPSS.

Significant correlations included:

1.Moderate:

a) IPP with prostate volume (p<0.0001; c=0.57), Figure-1;

b) IPSS and NQoL (p<0.0001; c=0.56), Figure-2.

2.Weak:

a) IPP and post-micturition residue (p <0.0001; c=0.31) and Qmax (p <0.0001; c=0.26);

b) IPSS and Qmax (p <0.0001, c=-0.21).

3.Very weak:

a) Age and IPP (p=0.003; c=0.14), Qmax (p=0.0003, c=-0.17) and IPSS-QoL (p=0.002, c=-0.14);

b) IPSS and post-micturition residue (p=0.0006; c=0.16) and PSA (p=0.04, c=0.14);

c) Prostate weight and age (p <0.0001, c=0.18).

## DISCUSSION

Since there are no measure to evaluate reliable clinical symptoms or specific and sensitive exams to evaluate the severity of bladder outlet obstruction (14), it is necessary to understand a series of correlated events and their potential pathophysiological disturbances.

There is no accepted universal “standardization” to diagnose and treat prostatic benign hyperplasia (BPH) with bladder outlet obstruction (BOO). Age, bladder perfusion, innervation and interactions of urothelium are responsible for LUTS and not only BOO (15-18).

Since clinical symptoms do not correlate with the grade of obstruction, at present, only urodynamic evaluation is the only objective method to diagnose BOO, and it is considered the gold standard method (17-21). However, urodynamics is an invasive method, expensive, that needs equipment not always available in different centers, and its routine use is improbable (20-23).

Physiologically, micturition is a complex phenomenum that depends on the interaction of several factors such as: apherent sensitive component, central nervous system (CNS), that coordinate these stimuli, muscle contraction and epherent component, including neurotransmitters; these components are not individualized but work together, and the weight of each one on micturition is variable; all these factors are responsible for the symptoms, and even men with obstruction may be asymptomatic (24).

BOO may present different heterogeneous signs and symptoms; clinical history is inaccurate for early diagnosis, and also, detrusor hypocontractility may be a differential diagnosis or also be present, since its clinical presentation is similar, with different pathophysiology and completely different treatment (25).

The most used tools to diagnose BPH and BOO include: complete medical history, IPSS and NQoL questionnaires, general physical exam and digital rectal examination, PSA, transabdominal prostatic ultrasound and free uroflowmetry.

In this study, IPSS did not correlate with age or prostate weight, but there was a weak negative correlation with maximum flow, and positive very weak with residual urine and PSA, since their characteristics are not exclusively related to BOO as shown in literature (26).

In order to improve the evaluation of quality of life it is possible to use other parameters such as productivity, sleep and vitality. In this matter, NQoL is a question form that does not quantify obstruction (the same as IPSS), but complements clinical evaluation of its impact on quality of life. In this study, IPSS and NQoL correlated moderately (p <0.0001; c=0.56), and the results were very similar to those of a Japanese study (p <0.0001; c=0.58) (27).

With ultrasound, it was observed a moderate correlation of IPP and prostate weight, and weak of IPP and residual urine and maximum flow, meaning that the higher the prostate volume, the higher the chance of IPP, in agreement with a previous study (6).

The study of free uroflowmetry is recommended due to its non-invasive nature, and it is used to screen and investigate BOO for a long time; however, although identifies patients with normal or low flow, it is not specific and does not differentiate low flow due to obstruction or hypocontractility.

Urinary flow results of a contraction of detrusor muscle against urethral resistance, and the loss of energy due to friction is 70% in men and 50% in women; therefore, low flow may be caused by lower contraction of detrusor muscle or higher urethral resistance, and maximum flow analysis may be inaccurate to diagnose BOO (23).

BOO due to BPH is related to mant more characteristics than mechanical obstruction, due to alterations of detrusor muscle, perfusion, expression of neurotransmitters at urothelium, that contribute to symptoms. This is the new concept of LUTS due to BOO secondary to BPH, related to many more hypothesis for understanding its pathophysiology (15, 17, 28).

A Chinese study corroborated our result, it described an evident overlapping of parameters used to evaluate LUTS. Despite significant correlations, it is not possible to predict the intensity of symptoms of obstruction, using isolated tools (29). Also, a Danish study observed statistically significant but weak correlations among non-invasive objective parameters during evaluation of LUTS (30).

Taken all together, these data support a complex correlation among the studied parameters, however IPSS shows a good correlation with patient’s perception of his quality of life, a main aspect to define and propose treatment and response evaluation in clinical trials (26).

Prospective evaluation with validated different and correlated tools in the same group of patients with LUTS, attended in a systemized manner in urological ambulatories, is important as demonstrated in the present study, that has also some limitations. This is a series of LUTS patients and the results can not be extrapoled to different scenarios, and not all patients had PSA values available.

Although the impact of nocturia on quality of sleep and life was evaluated, in urological researches this is an aspect not much studied. Our study is the second to correlate IPSS and NQoL (27).

Future studies are needed to confirm and broad the current results and should include urodynamics studies, although invasive when compared to the used tools, in order to evaluate in detail bladder function, and to classify patients with hyperactive bladder, urinary incontinence and with obstruction.

## CONCLUSIONS

In this study, we have found moderate, weak and very weak correlations among several parameters used to diagnose LUTS with BOO due to BPH. Since the value of these parameters is variable, particularly when the symptoms are mild, the creation of a logic and objective algorithm in the beginning and monitoring of treatment was not possible, and at present it is still based on interpretation of clinical symptoms.
